# Management of iatrogenic full thickness electrical burn in a preterm neonate using W-plasty technique combined with a median sternotomy incision

**DOI:** 10.1308/003588412X13373405387212

**Published:** 2012-05

**Authors:** E Chipp, H Duncan, R Papini

**Affiliations:** Birmingham Children’s Hospital NHS Foundation Trust,UK

**Keywords:** Electrical burn, Surgery, Neonatal, Iatrogenic, Reconstruction

## Abstract

Burns in the neonatal period are rare and most commonly due to iatrogenic causes. We report a case of a preterm neonate who sustained a full thickness electrical burn following the use of a temporary pacing pad. The case was complicated by significant co-morbidities and the need for cardiac surgery. We describe the surgical management of the case, using excision and closure in the form of a W-plasty. We discuss the reasons for this surgical decision and the importance of managing complex cases such as this on an individual basis.

Burns in the neonatal period are rare and commonly iatrogenic. Thermal injuries have been reported from alcoholic skin preparations, warming blankets, hot water bottles and diathermy pads.^1,2^ We present a case of a full thickness electrical burn due to the use of external pacing in a preterm neonate. This was managed successfully by combining excision and closure with a W-plasty plus a median sternotomy for cardiac surgery access.

## Case history

A female infant weighing 1.1kg was born at 34 weeks’ gestation by emergency Caesarean section due to foetal bradycardia and worsening hydrops. There was a prenatal diagnosis of congenital heart block and structural heart disease.

Initially, the heart rate of 70–90 beats per minute provided adequate cardiac output. However, at 2 days old, the baby had a heart rate of 30–45 beats per minute associated with hypotension unresponsive to medical treatment. Intravenous pacing was unsuccessful so a decision was made to perform transthoracic external cardiac pacing before performing definitive cardiac surgery and inserting pacing wires.

Paediatric transcutaneous pacing pads were placed on the anterior chest and back of the infant. Good capture was achieved and demand pacing was commenced with an immediate improvement in cardiac output. The pads were removed temporarily to perform echocardiography after approximately nine hours. At this point it was noted that there were several burns under the pads: three full thickness burns along the left sternal edge as well as a smaller full thickness burn on the left lateral chest wall and a partial thickness burn on the back.

The infant was due to undergo cardiac surgery via a median sternotomy approach later that day. It was felt that the proximity of the burns to the proposed surgical site would carry a significant risk of wound breakdown or mediastinitis from a potentially colonised burn wound. For this reason, the decision was made to excise the burn at the same time as the cardiac procedure.

**Figure 1:**
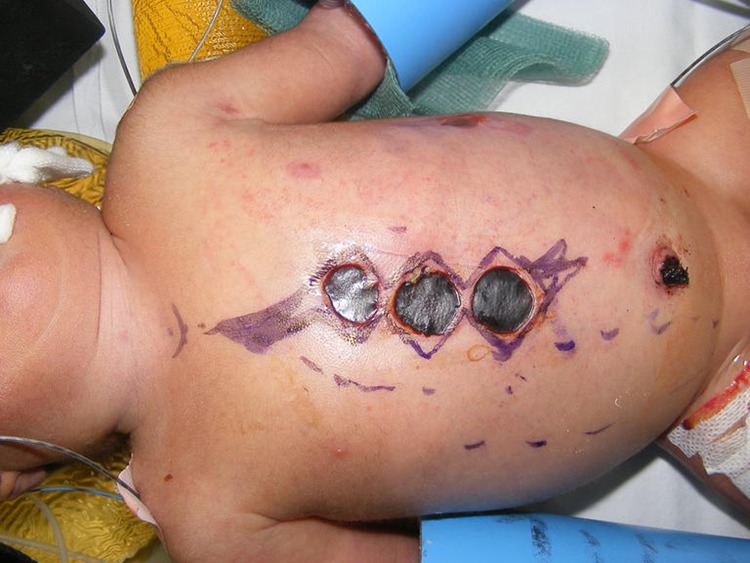
Pre-operative markings showing plan to excise burns as W-plasty, preserving small intact skin bridges

**Figure 2:**
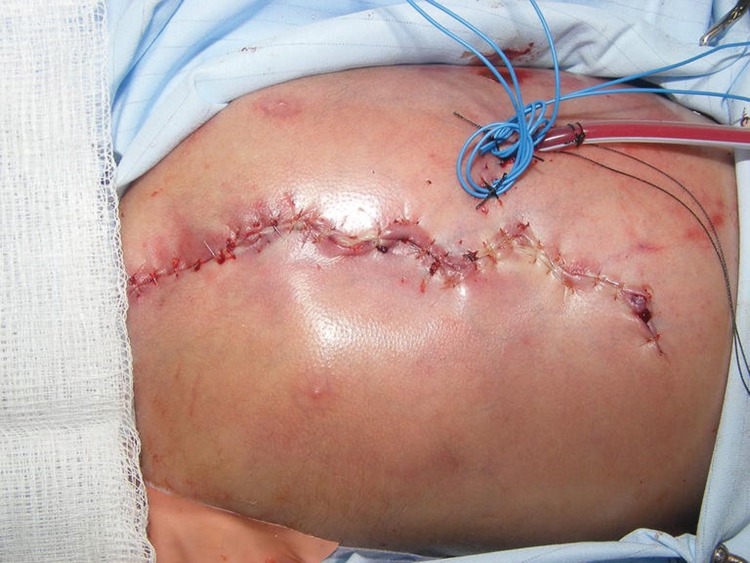
Post-operative view showing burns excised and closed as W-plasty. Drains and pacing wires visible on left side of chest to preserve blood supply to contralateral rectus muscle.

A median sternotomy was performed to allow access for ligation of a patent ductus arteriosus, pulmonary artery banding and insertion of pacing wires. The three circular electrical burns were excised as part of the incision (Fig 1). The burns were found to be full thickness and extended into the underlying pectoralis major and rectus abdominis muscle. The burns were excised in the form of a W-plasty, preserving the intact skin bridges in between the burns for wound closure. The skin edges were undermined for approximately 1cm each side in a subfascial plane to allow closure with minimal tension. After closure of the sternum and placement of a drain, the wound was closed in the form of a W-plasty with absorbable sutures (Fig 2).

## Discussion

Transthoracic cardiac pacing is an established treatment for conduction defects including congenital heart block. There have been previous reports of full thickness burns in neonates as a result of pacing pads similar to the ones used in this case.^3,4^ It is recommended that pads are moved to other sites on a regular basis but this can be difficult to achieve in preterm neonates with a small body surface area in relation to the size of the pacing pad. In addition, it is recommended that the skin surface is inspected regularly to check for signs of thermal damage.

This case illustrates the potential for significant morbidity to be caused inadvertently during potentially life-saving treatment. We highlight the importance of close monitoring of all infants who are receiving treatment with a risk of thermal injury.

Burns in the neonatal period are rare and commonly iatrogenic.^2,5^ Neonates have thinner skin than older children, meaning that burns are likely to be deep and may involve underlying structures. Management is complicated by physiological differences including an immature immune system, a large body surface area and relatively large fluid requirements. As many iatrogenic neonatal burns occur in an intensive care setting, many patients will also have significant underlying co-morbidities.

In such small infants it is vital to debride wounds accurately and preserve even small areas of undamaged skin. It is important to consider all available options from the reconstructive ladder and choose the option most suitable for the individual patient. In this case it was not possible to excise and close the burn as an ellipse without undue tension on the wound and a risk of wound dehiscence. Furthermore, it was felt that direct closure would have led to unacceptable medialisation of the nipple areola complex as well as leaving a vertical presternal scar in a patient at high risk of hypertrophic or keloid scar formation. Similarly, a skin graft would have led to an unsightly and potentially contracted and hypertrophic scar, in addition to the additional morbidity of a donor site.

**Figure 3:**
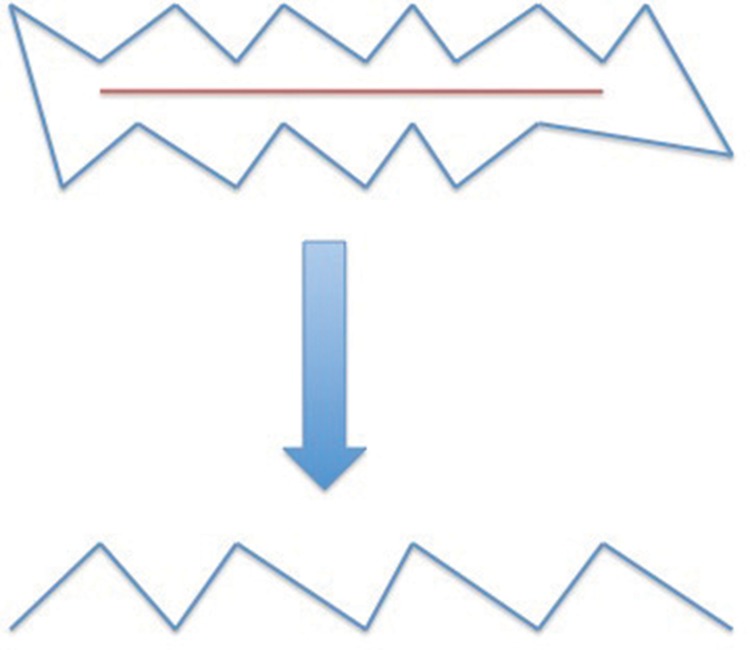
Planning a standard W-plasty: the markings to excise a linear scar (top) and the appearance of the wound when it is closed (bottom)

W-plasty is a surgical technique that is most commonly used to break up linear scars into multiple smaller ones, reducing tension and improving the appearance of the scar. It can also be used to help reorientate scars along relaxed skin tension lines. The name is due to the fact that the pre-operative and post-operative appearance resembles the letter W. It is designed by creating multiple small triangles along the length of a scar matched by a second row of triangles with points facing the bases of the triangles on the opposite side (Fig 3). In this case, the triangles were excised either side of the circular burn wounds rather than along a linear scar.

The decision to excise three separate burns as a W-plasty meant that the small intact skin bridges between the burns could be preserved, allowing direct wound closure. The W-plasty closure minimised the distortion of the nipple areola complex and avoided a vertical scar. It was predicted that future breast growth would be affected and this may require later reconstruction. For this reason, surgical drains and pacing wires were brought out through the left (injured) side of the chest, in order to preserve the blood supply to the contralateral rectus abdominis muscle in case this was required for later reconstruction. This illustrates the point that careful choice of procedure in the acute phase can expand reconstructive options in the future.

## Conclusions

Neonatal burns are rare, potentially complex and should be managed on an individual basis. This case illustrates the fact that not all burns will be suitable for standard tangential excision and grafting, and it may be necessary to incorporate other plastic surgical techniques to manage such cases. We have presented a case of successfully managing a full thickness electrical burn in a two-day-old preterm neonate using a surgical method, which has not been reported previously for this indication.
